# Hogs sleep like logs: Wild boars reduce the risk of anthropic disturbance by adjusting where they rest

**DOI:** 10.1002/ece3.10336

**Published:** 2023-07-22

**Authors:** Gustave Fradin, Simon Chamaillé‐Jammes

**Affiliations:** ^1^ CEFE, Univ Montpellier, CNRS, EPHE, IRD Montpellier France

**Keywords:** activity pattern, anthropization, habitat selection, human activities, sleep

## Abstract

Many animals living in anthropized landscapes try to avoid encountering people by being active at night. By doing so, however, they risk being disturbed while at rest during the day. To mitigate this risk, diurnally resting species may be highly selective about where they rest. Here, we used GPS and activity sensors to study how wild boars (*Sus scrofa*) might adjust their resting site selection and revisitation patterns to the risk of disturbance by people. We evaluated the probability of daytime relocation to assess the efficacy of wild boars' resting strategy in reducing the risk of human encounter while at rest. We attempted to identify the cause of some relocations using audio recordings. Generally, we found that wild boars did not specifically avoid resting near villages or roads, that is, where the risk of encountering people is higher, if they could find sites with suitable vegetation cover. The risk of disturbance by people was low, even near villages. Resting sites located close to villages were visited more repeatedly than those located further away, suggesting that focusing on a few familiar and quiet resting sites was a successful strategy for resting undisturbed in an anthropized landscape.

## Graphical abstract

The selection of a resting site is a daily decision with possibly long‐lasting implications for animals, but how human activity affects this aspect of their lives is not well understood. Using tracking methods, we studied how wild boars mitigate the risk of disturbance by people while resting. We found that they can safely rest very close to humans by revisiting well‐known and safe resting sites.

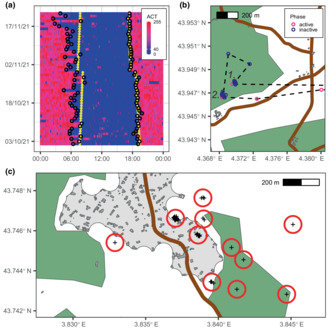

## 1 INTRODUCTION

Resting is a crucial daily requirement for all animals. It serves various purposes such as energy conservation (Glass et al., 2021; Riede et al., 2017), thermoregulation (Lutermann et al., 2010), and predator avoidance (Lima et al., 2005). Additionally, rest encompasses sleep, which is essential for neuro‐physiological homeostasis (Freiberg, 2020; Schmidt, 2014). To meet physiological requirements and integrate ecological constraints, such as predation risk, animals allocate a significant proportion—often more than half—of their daily time budget to resting, which takes place at specific resting sites (Campbell & Tobler, 1984; Siegel, 2009; Ungurean et al., 2020). These sites may vary in quality, and in particular may offer different levels of safety (Burger et al., 2020; Markham et al., 2016). Thus, resting site selection can be fine‐tuned to minimize the risk of predation or disturbance, and the need for daily rest is likely to have an important influence on how animals use the landscape.

In many environments, animals are facing increasing anthropic pressures that can impact their resting behavior (Gaynor et al., 2018; Wilson et al., 2020). It is well documented that many species modify their activity patterns in response to human pressures and tend toward nocturnality to avoid being active during the day, when the intensity of human activity is highest (Gaynor et al., 2018). Such a response to anthropization, however, exposes resting behavior to higher risks of disturbances by people. When at rest, animals may be subjected to both targeted (e.g., hunting) and untargeted (e.g., outdoor sports) disturbances and could be expected to adjust their resting strategy—and in particular where they rest—to minimize the risk of being disturbed. Indeed, some species have been shown to fine‐tune their selection of resting sites to the perceived level of risk of anthropic disturbances. For instance, wolves have been demonstrated to select more concealed resting sites after an encounter with people (Wam et al., 2012), and to proactively choose to rest farther from human infrastructures (Bojarska et al., 2021). Similarly, elephants have been observed returning more often to preferred resting sites, when ranging outside protected areas (Wittemyer et al., 2017). However, despite a few documented examples such as these, our overall understanding of adjustments of resting site selection and revisitation rate to the risk of disturbance by people remains limited. Additionally, we are not aware of any study having quantified the actual success of such adjustments in reducing the likelihood of being disturbed. Generally, there is very little information about how frequent anthropic disturbances of resting animals are in the wild.

One species that offers great opportunities to learn more about these adjustments is the wild boar (*Sus scrofa*). It is a widespread species that can be found to survive and exploit even the most anthropized landscapes (Stillfried et al., 2017). When living near people, wild boars could naturally be subjected to non‐targeted disturbances (e.g., by walkers and dogs being walked). Additionally, as a commonly favored game species, wild boars are subject to hunting. Wild boars are known for their great behavioral flexibility and are able to adjust their patterns of activity to various factors including the season, the food availability (Brivio et al., 2017; Keuling et al., 2008), but also the risk of human encounter (Johann et al., 2020; Ohashi et al., 2014; Rosalino et al., 2022). Although they can be readily active during the day where human densities are low (Podgórski et al., 2013), they become more nocturnal when they live near people (Ikeda et al., 2019; Podgórski et al., 2013), and when they are hunted (Johann et al., 2020; Keuling et al., 2008). Most outdoors activities—including recreational hunting—occur during the day. By switching to a predominantly nocturnal pattern of activity in human‐dominated landscapes, wild boars chose to expose their resting phase to a higher risk of anthropic disturbance. This likely is a successful strategy for risk mitigation, as resting is a well‐known and efficient anti‐predator strategy (Lima et al., 2005). However, this also means that the daily choice of a resting site is critical for minimizing the risk of encountering people. When nocturnal, wild boars are monophasic sleepers and usually remain at rest for the entire daytime period. This implies that the choice of a specific resting site, made in the early morning, determines the exposure of the animal to the risk of disturbance for the rest of the day. The decision‐making process for choosing a resting site has several components, offering several opportunities to adjust to the risks imposed by people. For example, wild boars could adjust where they rest in the landscape (e.g., distance to infrastructures, habitat types), and how they use their network of potential resting sites, coming back to some more often. It is well known that anthropic pressures can shape the spatial behavior of wild boars (Stillfried et al., 2017; Tolon et al., 2009), and several studies have investigated how their use of resting sites varies with the risk of being hunted (Maillard & Fournier, 1995; Saïd et al., 2012; Scillitani et al., 2010; Sodeikat & Pohlmeyer, 2007). However, studies rarely considered the risk of disturbances unrelated to hunting, despite their potential importance in anthropized landscapes (Marzano & Dandy, 2012).

Here, we present a study of the resting strategy of wild boars living in a rural Mediterranean landscape in the South of France. Using activity and GPS data collected on collared animals, we examined how wild boars modify their resting site selection in response to the risk of anthropic disturbance. This includes the assessment of (i) the potential avoidance of areas where meeting people is more likely (i.e., close to villages and roads), and (ii) the selective re‐use of previously visited sites, depending on their location in the landscape with regard to the risk of being disturbed. We also estimated, across the landscape, (iii) how frequently wild boars engaged in a significant and potentially costly relocation to a secondary site, and attempted, when possible, to identify the cause of these relocations using audio recordings. Overall, our study provides insights into the effectiveness of behavioral adjustments in reducing the risk of disturbance of rest by animals living in anthropized landscapes.

## 2 MATERIALS AND METHODS

### 2.1 Study sites

We studied wild boars in the *Gorges du Gardon* (43.93° N; 4.38° E) and the *Pic Saint‐Loup* areas (43.74° N; 3.88° E), in southern France. Both sites are very similar, the landscape forming a mosaic of agricultural lands, densely vegetated patches (shrublands and forests), and villages, covering 38%, 48%, and 11% of the land in the *Gorges du Gardon* area, and 55%, 29%, and 12% in the *Pic Saint‐Loup* area, respectively. The altitude is ~100 m. Vineyards make up most of the agricultural lands. The natural vegetation patches are mostly Mediterranean *garrigues*, with thick understory and dominated by dense evergreen oak (*Quercus ilex*) or Aleppo pine (*Pinus halepensis*) tree stands. Villages have hundreds to a few thousand inhabitants, and the general population density is approximately 280 inhabitants/km^2^, with some isolated houses also spread throughout the landscape. Both sites are located about 10 km away from a large city (Nîmes for the *Gorges du Gardon* area, Montpellier for the *Pic Saint‐Loup* area). Wild boars are mostly hunted in drive hunts involving an average of 20 hunters (usually between 10 and 40) and about three to 15 hounds. The open season for drive hunt lasts from the 15th of August to the end of February or March, depending on the year and the study area. In addition to the risk induced by hunting, wild boars could potentially be disturbed, at both study sites, by agricultural and recreational outdoors activities (e.g., biking, hiking, dog‐walking, etc.). The lack of natural predators in the study areas guarantees that human activity represents the only potential risk of disturbance for resting wild boars. Part of the *Gorges du Gardon* area is a densely vegetated military zone where public access is denied, although recreational hunting of wild boars is allowed. The military activities, however, are likely to represent a source of disturbance for the wild boars, even though we could not evaluate how the level of human disturbance could differ within and out of the military zone.

### 2.2 Captures and tracking

**FIGURE 1:**
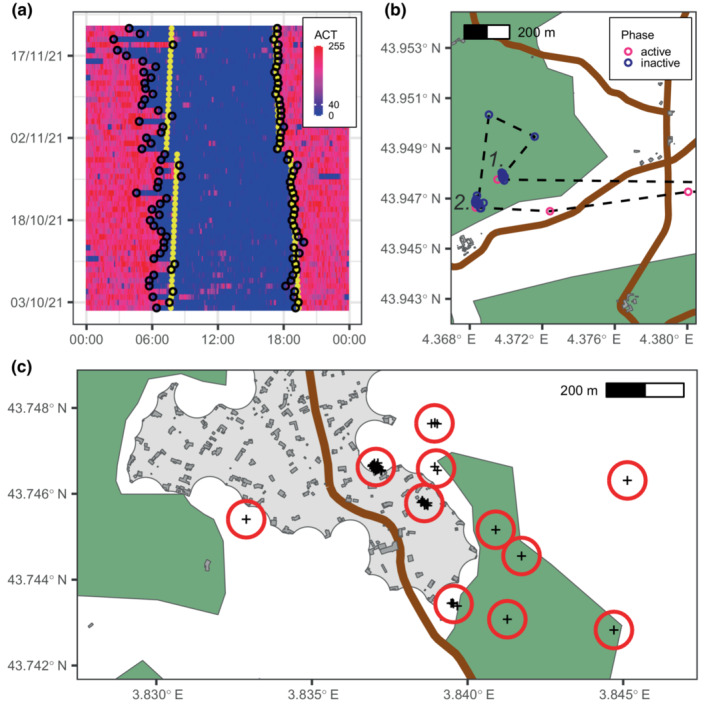
(a) Example of the activity pattern of a wild boar. The color codes for the value of the ACT variable (blue = low activity; red = high activity). The black circles indicate the start and end times of the inactive phases. The yellow dots indicate the times of sunrises and sunsets. (b) Example of a wild boar's relocation during an inactive phase. The black dashed line represents the trajectory linking GPS fixes (pink and blue circles). The animal first rested in (1) and then relocated to spend the rest of the inactive phase in (2). (c) Example of the resting site revisitation pattern of a wild boar. The daily resting locations (black crosses) are clustered into resting sites (red circles). In (b) and (c), buildings are represented in dark gray, villages in light gray, roads in brown, and densely vegetated patches in dark green.

From 2018 to 2022, we captured wild boars with corn‐baited traps, using a protocol approved by the ethical committee of the French Ministry of Research (APAFIS#20279‐2019041522576537v3). We immobilized them either with a small immobilization box, or through anesthesia, using a combination of tiletamine (3 mg/kg), zolazepam (3 mg/kg) and xylazine (0.3 mg/kg), and using atipamezol (0.03 mg/kg) to reverse xylazine at the end of the operations. We equipped 22 animals weighing more than 40 kg with a GPS collar. The collars acquired a GPS location every 30 min, and a measure of activity level (hereafter ACT) every 5 min. ACT was calculated as the average of 8 Hz acceleration measurements over three orthogonal axes and was available to us on a dimensionless scale ranging from zero (indicating no movement of the collar over the 5‐min interval) to 255. As values were highly correlated between axes, we only used the ACT data corresponding to the anteroposterior axis. We recovered two collars after the animals had been killed by a collision with a vehicle, six after the animals had been shot by local hunters, nine through the activation of the collars' remote drop‐off system, and five after they had fell off the animals prematurely. Out of these five collars, three dropped less than 15 days after deployment and we excluded the corresponding animals from the study. After a capture, a wild boar can display reduced activity levels for several days (Brogi et al., 2019). After visual inspection of the patterns of activity, we chose to exclude the first 3 days following each capture from the analyses, which resulted in survey periods averaging 126 days long (SD = 53). The activity profiles of the animals clearly showed that most of them were nocturnally active, and rested each day across the daylight hours, in one consolidated bout (example in Figure 1a). Only two animals were significantly active during the day during parts of their survey period and were excluded from the analysis to focus only on daytime resting strategies. 17 animals (12 males and five females) remained in our final dataset. Two males and two females had been using extensively the military zone. No pair of collared animals moved together as part of the same sounder. Due to the very limited movement data from females, we excluded them from the analyses. As male and female wild boars generally have different spatial ecology (Morelle et al., 2015; Saïd et al., 2012), this avoided a possible bias in our analyses, which can be interpreted as describing the behavior of male wild boars. Note, however, that exploration of the female data did not suggest that their behavior differed largely from that of males, with regard to aspects investigated here (Appendix S1). Also, for brevity, we hereafter refer to the animals studied in the analyses as “wild boars,” without specifying their sex each time.

### 2.3 Data preparation

#### 2.3.1 When did resting occur?

To study where wild boars rested, we first needed to determine when they rested each day. Upon visually examining their activity patterns (Figure 1a), it was evident that wild boars consistently divided their 24‐h cycle between an inactive phase (primarily resting with some occasional activity) and an active phase (during which they could occasionally rest). To recover the start and end times of these inactive phases, we first classified each 5‐min period as “resting” (ACT ≤ 40) or “active” (ACT > 40; see Appendix S2 for details). This allowed us to identify a continuous inactive phase spanning the daytime hours each day, separated from the previous and next inactive phases by an active phase. During this process, we disregarded the short bouts of activity that occurred between long‐lasting “resting” bouts, which were irrelevant for the description of the large‐scale pattern of activity. The resulting patterns closely matched those anticipated based on visual inspection (see Appendix S2).

#### 2.3.2 Where did resting occur?

We used the GPS data to determine where the wild boars rested during each inactive phase. Usually, the entire inactive phase was spent at a single location. On some occasions, however, the wild boars would start resting in one place and then move to another location to spend the rest of the inactive phase (example in Figure 1b). To identify such relocations, we looked for any substantial movements recorded in the ACT data during the inactive phases. We compared the mean GPS position of the animal before and after the high ACT values, and if those locations were more than 100 m apart, we considered that the animal had relocated. See Appendix S2 for more details.

Throughout their lives, wild boars tend to return to rest at sites where they have rested before. To test whether this behavior could be affected by where the animals rested in the landscape, we first had to identify where their resting sites (hereafter called RS, example in Figure 1c) were located. We did so by calculating the mean of the GPS positions of each inactive phase. We ignored in this process the GPS locations acquired after a relocation happened, so that the positions of the RSs reflected the choices made by the wild boars at the end of their active phase, to select a place where to settle for the day. We then performed a hierarchical clustering analysis on these mean GPS positions, using an average linkage, to define clusters that were more than 50 m apart. All the locations that fell within the same cluster were considered to be revisitations of the same RS.

#### 2.3.3 Environmental variables

Our study sites could be coarsely partitioned between villages, agricultural lands, and densely vegetated areas. Wild boars rarely rested inside villages, and when they did so, they always stayed at the outskirts, in the transitional zones between villages and agricultural lands. To simplify, we characterized the vegetation cover of each RS as a binary variable, indicating whether it was located within a densely vegetated patch or not, at a large scale. We used the CORINE Land Cover (2018) data to identify large patches of dense vegetation, that we distinguished from villages and agricultural lands scattered with small thickets and hedgerows. We considered that a wild boar could rest either inside or outside densely vegetated patches. See Appendix S3 for more details.

Then, we used OpenStreetMap (OSM) to calculate the distance of each RS to two features associated with higher risks of human disturbance: villages and roads. We laid out the road network by combining the “motorway,” “trunk,” “primary,” “secondary,” and “tertiary” road categories from OSM. This way we excluded the residential roads, the roads with very low traffic, and the forest tracks, along which anthropic activity was infrequent. We also used OSM data to determine the edges of villages. To do so, we applied a buffer of 50 m around OSM's polygonal layer for buildings and merged the polygons that overlapped after this operation. This allowed us to identify villages as clusters of buildings closer together than 100 m. We then shrunk the obtained polygons with a buffer of −50 m, so that their edges matched the edges of the villages' peripheral buildings. Any such polygon larger than 1.5 hectares was considered a village and visual inspection confirmed the adequacy of this approach. We calculated the distances of each RS to the nearest road, and village edge (with negative distances indicating locations inside villages).

Finally, we associated each visit of a RS to the status of the hunting season the day it occurred. Drive hunting is the primary hunting technique for wild boars in the study areas, and the only one that targets resting animals. Therefore, we defined the hunting season (HS) based on the dates of the open season for drive hunting (see section 2.1). Drive hunts were only permitted on “hunting days” (Wednesdays, Saturdays, Sundays, and bank holidays). During the non‐hunting season (NHS), drive hunts were prohibited, except with a specific authorization from the local authority, concerning a negligible number of hunts. We did not account for the COVID‐19 lockdowns because they never affected the hunting patterns during the periods in which we monitored the animals. Although lockdowns may have reduced the probability of untargeted disturbances, in our study this only affected 28 days for four animals.

### 2.4 Data analysis

#### 2.4.1 Habitat selection for resting sites

We investigated how the selection of RSs by wild boars varied with vegetation cover and proximity to villages and roads. We estimated this selection within the home ranges of the animals, defining the home range as the contour of the 90% utilization distribution, estimated with a standard kernel density approach, from GPS locations collected during the active phase. For each used RS of each wild boar, we drew 1000 random locations in the wild boar's home range and considered these locations to be available RSs. For both used and available RSs, we extracted the dominant vegetation type (open = 0; densely vegetated = 1) and the distance to roads or villages. We then fit a standard resource selection function (RSF) using a generalized linear mixed model (GLMM), using the type of location (used vs. available) as response variable and a binomial distribution for errors. Predictor variables included an interaction between vegetation type and distance to roads and an interaction between vegetation type and distance to villages. We also included individual animal identities nested within study sites as random intercepts. We also expected the hunting season to influence how wild boars selected RSs. However, a non‐negligible proportion of the RSs had been visited during both the HS and the NHS (10% of all RSs), so that we could not simply include the hunting season in the RSF as a predictor variable. We thus decided to run two separate models: one with the RSs visited at least once during the HS, and one with the RSs visited at least once during the NHS. We then calculated selection ratios as a metric of habitat selection following Chamaillé‐Jammes (2020). A selection ratio above one indicates selection, and a value below one indicates avoidance. We estimated the fit of the RSF models using the now well‐established k‐fold cross‐validation approach proposed by Boyce et al. (2002). This approach uses the Spearman‐rank's correlation as the model's performance metrics. For each model, we used 5 folds, 10 bins of RSF scores, replicated the calculations 20 times and reported the average Spearman‐rank correlation and its standard error. See Boyce et al. (2002) for details on the approach.

#### 2.4.2 Resting sites' revisitation rate

Some RSs were preferred and visited more often than others. We investigated how the proximity of villages and roads influenced the frequency of visits to RSs. We did so by fitting a GLMM on the number of visits to each RS, using a zero‐truncated Poisson regression that contained the same predictors as our RSF analysis: the vegetation cover in interaction with the distances to roads, and to villages. The length of the survey period could naturally influence the number of times a RS was visited (but does not have to, as a wild boar could also use new RSs as the survey progresses), and we therefore included this variable (log‐transformed to account for the link function of the Poisson regression) in the model as a control variable. Finally, we included individual animal identities, nested within study sites, as random intercepts. As for the habitat selection analysis, we fitted one model for the NHS and one for the HS. The marginal Nakagawa's *R* ^2^ was used as a measure of model fit (Nakagawa & Schielzeth, 2013).

#### 2.4.3 Relocation events

We used relocation events to gain insight into how often wild boars are disturbed while at rest. We modeled the probability of a wild boar to relocate as a binary (0 = no relocation; 1 = relocation) response variable using a binomial GLMM. The predictor variables were an interaction between vegetation type and the distance to roads, an interaction between vegetation type and the distance to villages, and a season variable accounting for the risk of being hunted. We expected that the relocation probability was influenced not only by the HS being open or closed, but also by whether the day was actually a hunting day or not. Therefore, we used a 3‐category variable coding for “HS and hunting day,” “HS and non‐hunting day,” and “NHS.” This differed from the analysis of habitat selection of RSs above, in which we implicitly studied proactive adjustments of resting behavior to human activity and assumed that there could be a seasonality linked to hunting in wild boars' behavior, without accounting for hunting days. In addition to the predictor variables, we included individual animal identities, nested within study sites, as random intercepts. We also modeled the distance traveled between RSs each time a relocation event occurred using a similar model and the same predictor variables. The marginal Nakagawa's *R* ^2^ was used as a measure of model fit.

#### 2.4.4 Audio data extraction and analysis

Six of the collars, deployed on four males and two females, had an audiologger (Latorre et al., 2021), that is, an autonomous microphone, which recorded an average of 16 days (SD = 9) of continuous audio data after capture. We used these data to gain insight into what exactly happened right before relocations occurred. In particular, we looked for sounds that could attest disturbances by people. Even though the females were excluded from the main analysis, we considered the audio data from both sexes, because we did not expect sex to influence the nature of the possible causes of relocations, and because the amount of relocation events for which we had audio data was low. Nine relocation events occurred during a period with audio monitoring, with an average distance traveled of 170 m (maximum = 317 m). For each relocation event, we precisely identified the moment the wild boar stopped resting (a sharp rise in ACT) and extracted the audio from 40 min prior to this moment to 5 min after. This way, we ensured that the extract started while the wild boar was resting and quiet and ended after it had started to relocate. For each of those “relocation” audio tracks, we also extracted one “control” track, recorded at the same time of day but on a different date. Tracks are available in Appendix S4. We randomly sorted all tracks, listened to them while viewing the corresponding spectrogram, and noted any sounds that could be identified, focusing specifically on sounds related to human activities (e.g., motor sounds, dog barks, human voices, gunshots) that could have interrupted the resting of wild boars. Of course, this approach might have missed some disturbances if they occurred silently or if the wild boars were able to detect danger and relocate before any sound could be picked up by the microphone. However, a sensitivity trial we conducted under realistic conditions suggested that the microphones were sensitive to even faint distant sounds: we placed a microphone in the vegetation, facing the ground, and spoke in a calm voice several meters away from it. We were able to identify normal speech both in the audio recording and in the spectrogram when the experimenter stood as far as 30 m away from the microphone, facing it (20 m when facing the opposite direction).

## 3 RESULTS

### 3.1 Timing and duration of inactive phases

The wild boars studied spent most of their time at rest. On average, 58.5% (SD = 4.4) of their time budgets corresponded to the inactive phase, which typically started at the last hours of the night and spanned throughout daytime (examples in Figure 1a; Appendix S2). Activity patterns were mostly synchronized with sunset times throughout the year. The end of the inactive phase occurred within 30 min around sunset in 44% of cases (and within 90 min in 80% of cases), whereas it started within 30 min around sunrise in only 24% of cases (and within 90 min in only 53% of cases).

### 3.2 Patterns of resting site selection

#### 3.2.1 Habitat selection for resting sites

**FIGURE 2:**
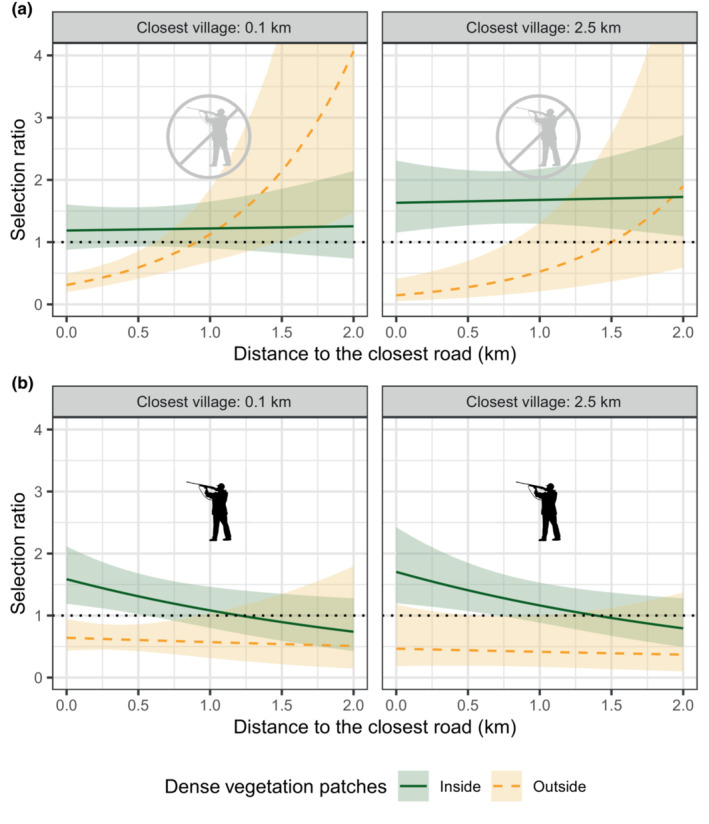
Estimation of the selection ratio for resting sites by wild boars during the non‐hunting season (a) and the hunting season (b), for different distances to roads and villages. Selection for densely vegetated patches is represented in a dark green, solid line. Selection for non‐densely vegetated patches is represented in a yellow, dashed line. A selection ratio above one indicates that sites are selected, and a selection ratio below one indicates that sites are avoided. Lightly colored ribbons show the 95% confidence intervals.

As expected, wild boars favored resting in dense vegetation patches and generally avoided resting at sites that were in more open habitats (Figure 2; Table 1). During the NHS, the proximity of roads or villages had no effect on the selection of sites by wild boars, as long as they were in dense vegetation (Figure 2a; Table 1). Sites in more open vegetation were avoided when they were close to roads, but this avoidance disappeared, maybe shifting to a selection, when they were far from roads (Figure 2a; Table 1). Note, however, the large confidence intervals, caused by the fact that little data were available for those sites, that are rare in the landscape. During the HS, wild boars also did not adjust their selection to the distance to the closest village (Figure 2b; Table 1). In contrast to the NHS, they, however, did select more strongly sites located close to roads when they were in dense vegetation (Figure 2b; Table 1). The proximity of roads had no effect on the selection of sites in more open vegetation, and they were always avoided in this season (Figure 2b; Table 1).

**TABLE 1:** Parameters and statistics for the models estimating the selection ratio for resting sites during the non‐hunting season and the hunting season.

Variable	*β*	SE	*z*‐Value	*p*‐Value
Model: Selection ratio for resting sites during the non‐hunting season				
Intercept	−8.04	0.24	−33.20	<.001
Inside densely vegetated patches	1.29	0.29	4.45	<.001
d(road)	1.28	0.31	4.10	<.001
d(village)	−0.32	0.24	−1.34	.18
Inside densely vegetated patches × d(road)	−1.26	0.35	−3.58	<.001
Inside densely vegetated patches × d(village)	0.45	0.25	1.77	.08
Model: Selection ratio for resting sites during the hunting season				
Intercept	−7.34	0.21	−35.44	<.001
Inside densely vegetated patches	0.89	0.26	3.46	<.001
d(road)	−0.12	0.37	−0.31	.75
d(village)	−0.13	0.21	−0.62	.53
Inside densely vegetated patches × d(road)	−0.27	0.40	−0.67	.5
Inside densely vegetated patches × d(village)	0.16	0.23	0.70	.48

*Note*: “d(village/road)” stands for “distance to the closest village/road.” The 5‐fold cross‐validation performance scores (Spearman‐rank correlations) of the models are, respectively, 0.61 ± 0.16 SE and 0.72 ± 0.14 SE.

#### 3.2.2 Resting sites' revisitation rate

**FIGURE 3:**
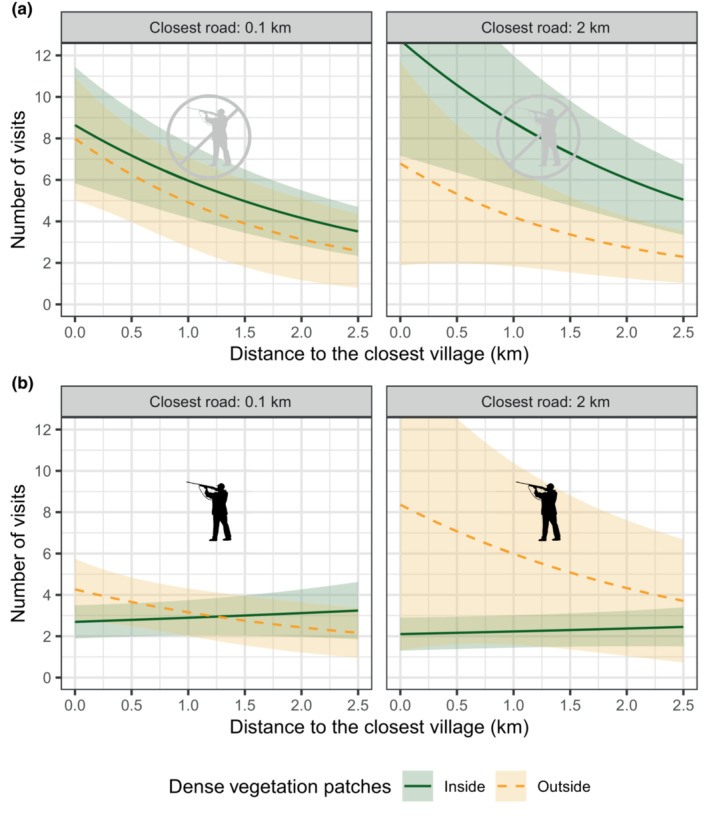
Estimation of the number of visits to resting sites over a 6‐month period, during the non‐hunting season (a) and the hunting season (b), for different distances to roads and villages. The estimation for the resting sites located inside densely vegetated patches is represented in a dark green, solid line. The estimation for the resting sites located outside those patches is represented in a yellow, dashed line. Lightly colored ribbons show the 95% confidence intervals.

Wild boars regularly revisited RSs (51% were visited more than once), and some were visited more often than others (Figure 1c). RSs located close to villages were frequently revisited during the NHS and significantly more often than those located further away, irrespectively of whether they were in dense vegetation or not (Figure 3a; Table 2). Except the control variable of the length of the survey period, no other predictors were influential on the number of visits to RSs, during the NHS (Table 2). During the HS, we found significant effects of the type of vegetation—and its interactions with the distances to villages and roads—on the number of visits to RSs during the HS (Table 2). Note, however, that the predicted number of visits to RSs remained low during the HS, except far from roads and outside densely vegetated patches, where the confidence intervals are large, due to limited data and little availability of the habitat (Figure 3b).

**TABLE 2:** Parameters and statistics for the models estimating the number of visits to resting sites during the non‐hunting season and the hunting season.

Variable	ß	SE	z‐Value	*p*‐Value
Model: Number of visits to resting sites non‐hunting season				
Intercept	−1.17	0.73	−1.60	.11
d(village)	−0.49	0.19	−2.57	.01
d(road)	−0.09	0.20	−0.43	.67
Inside densely vegetated patches	0.05	0.15	0.32	.75
Log(length of the survey period)	0.63	0.16	3.88	<.001
Inside densely vegetated patches × d(village)	0.12	0.20	0.59	.55
Inside densely vegetated patches × d(road)	0.29	0.21	1.36	.17
Model: Number of visits to resting sites hunting season				
Intercept	−0.40	0.64	−0.63	.53
d(village)	−0.34	0.18	−1.85	.06
d(road)	0.36	0.22	1.64	.1
Inside densely vegetated patches	−0.48	0.17	−2.77	<.01
Log(length of the survey period)	0.35	0.14	2.41	.02
Inside densely vegetated patches × d(village)	0.43	0.20	2.11	.03
Inside densely vegetated patches × d(road)	−0.55	0.26	−2.12	.03

*Note*: “d(village/road)” stands for “distance to the closest village/road.” The model's *R* ^2^ are, respectively, 80% and 39%.

### 3.3 Relocations

**FIGURE 4:**
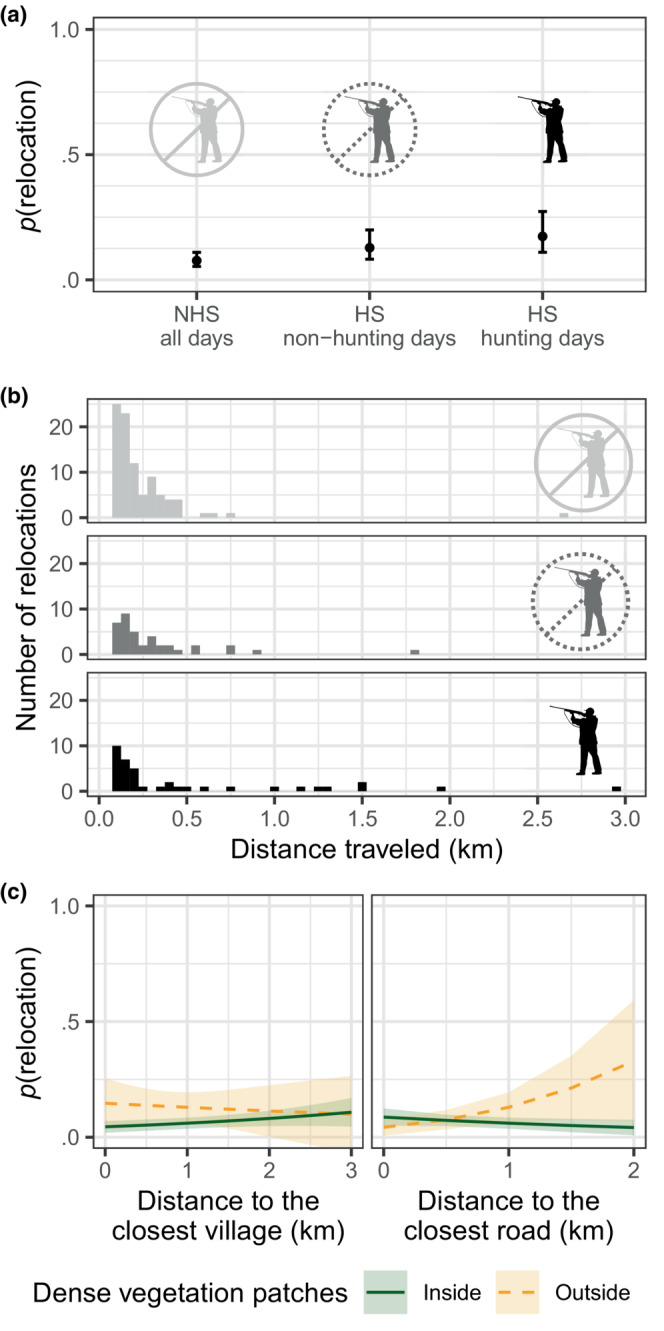
(a) Estimation of the probability of relocation during the hunting season and hunting days (Wednesdays, Saturdays, Sundays, and bank holidays), during the hunting season and non‐hunting days, and during the non‐hunting season. (b) Distribution of the distances traveled during relocations, according to hunting periods (same categories as in (a)). (c) Estimation of the probability of a relocation, during the non‐hunting season, according to the distance to the closest village and to the closest road. The estimation for the resting sites located inside densely vegetated patches is represented in a dark green, solid line. The estimation for the resting sites located outside those patches is represented in a yellow, dashed line. Lightly colored ribbons show the 95% confidence intervals.

The probability that a relocation occurred during the inactive phase was very low during the NHS (0.08; 95% CI [0.05–0.11]; Figure 4a; Table 3). Although the probability of relocation remained relatively low in the HS, it did increase twofold during the hunting days of the HS (0.17; 95% CI [0.11–0.27]; Figure 4a; Table 3). Besides, when a relocation happened during the NHS, the distance traveled was virtually always less than 500 m (Figure 4b). Indeed, almost all the few long‐distance relocations (17 out of 21 of the relocations >500 m) happened during the HS (Figure 4b). When the animal was initially resting in dense vegetation patches, neither the distance to the closest village, nor the distance to the closest road affected the probability of a relocation (Figure 4c; Table 3), or the distance traveled on relocations when they occurred (Table 3). When the animal was initially resting outside dense vegetation patches, there were a few significant effects of distance to the closest village or road on the probability of relocation and on the distance traveled, that were likely not biologically relevant (small effect or large confidence interval) and possibly caused by the low sample size in this condition (Table 3). Note however that the *R* ^2^ of both models were very low (Table 3), indicating that a large part of the variability in both the probability of relocation and the distance traveled on relocations was not explained, neither by hunting nor by the other variables considered in the models.

**TABLE 3:** Parameters and statistics for the models estimating the probability and the distance of relocations.

Variable	*β*	SE	z‐Value	*p*‐Value
Model: Probability of relocation				
Intercept	−2.96	0.34	−8.66	<.001
d(village)	−0.15	0.41	−0.36	.72
d(road)	1.20	0.44	2.69	<.01
Inside densely vegetated patches	0.29	0.37	0.77	.44
During HS out of hunting days	0.51	0.25	2.08	.04
During HS and hunting days	0.82	0.25	3.25	<.01
Inside densely vegetated patches × d(village)	0.46	0.43	1.07	.29
Inside densely vegetated patches × d(road)	−1.59	0.51	−3.09	<.01
Model: Distance of relocation (km)				
Intercept	−1.44	0.25	−5.77	<.001
d(village)	0.51	0.27	1.92	.05
d(road)	−0.41	0.28	−1.48	.14
Inside densely vegetated patches	−0.23	0.25	−0.92	.36
During HS out of hunting days	0.18	0.15	1.16	.25
During HS and hunting days	0.54	0.17	3.22	<.01
Inside densely vegetated patches × d(village)	−0.42	0.28	−1.50	.13
Inside densely vegetated patches × d(road)	0.71	0.33	2.18	.03

*Note*: “d(village/road)” stands for “distance to the closest village/road.” The models' *R* ^2^ are, respectively, 6% and 16%.

In all the nine audio tracks extracted from before relocations and the nine control tracks, the wild boars could be heard resting quietly, usually snoring, breathing calmly, and grooming. Although we could hear vehicles passing at close distance in two control tracks, the wild boars did not seem to react to those sounds. In eight extracts corresponding to a relocation, we could hear the wild boars walking away, without any disturbance being identifiable in the audio. In the ninth extract, which was recorded on a Sunday during the HS, we could clearly identify a hunting event by the barks of a pack of dogs and a gunshot, right when the wild boar started to move away (Appendix S4). We did not hear any other potential disturbance linked to human activity in the selected extracts.

## 4 DISCUSSION

Resting is an important part of an animal's life, both in duration and function. Yet, our knowledge of the flexibility of animals' resting behavior remains limited. In particular, we know little about how animals adjust their resting behavior to the risk of human disturbances, how often actual disruptions of resting phases occur, and how animals respond to them. The wild boars we studied allocated more than half of their time budget to resting, mostly during daytime, and thus preferred to be exposed to anthropic disturbances when resting compared to when foraging. This pattern has been observed in other populations of wild boars (Brivio et al., 2017; Johann et al., 2020; Rosalino et al., 2022) but also in a variety of other species (Gaynor et al., 2018) living in anthropized landscapes. We questioned whether wild boars tried to minimize the risk of being disturbed through resting site (RS) selection, and in such a case, whether they were successful.

As expected, wild boars always favored resting under the cover of densely vegetated patches, which is certainly the best way to reduce the risk of being disturbed. Additionally, we expected the selection of RSs to vary with the risk of encountering people. Surprisingly, however, we did not find any effect of the proximity of villages on the selection of RSs. As resting near villages is undoubtedly associated with costs and benefits, linked to both resource acquisition and safety, these must probably offset each other so that there is no net advantage of selecting to rest near or far from a village. The fact that the proximity of villages did not affect habitat selection for RSs however contrasted with the strong effect it had on the fidelity to RSs, during the NHS. When they were not at risk of being hunted, wild boars came back to rest at the same spot more often when it was close, or even within, a village. We tentatively suggest that predictably quiet places, suitable for resting, are rare near villages, and that wild boars rely heavily upon them. This effect disappeared during the HS, as revisitations of RSs were less frequent, maybe because wild boars avoid being predictable when they are actively being sought by hunters. Our study shows that the need to find suitable RSs does not prevent wild boars from exploiting areas with high degrees of proximity with people, as they can compensate a general increased probability of disturbance by selecting more intensely some specific RSs that they know are safe. This plasticity in wild boars' resting strategy is certainly critical to explain their success in subsisting, and sometimes thriving, in a wide diversity of habitats, even those heavily urbanized (Stillfried et al., 2017).

The effect of roads on wild boars' RS selection was more complex. During the NHS, wild boars selected to rest in densely vegetated patches irrespectively of their distance to roads. In contrast, wild boars avoided resting outside densely vegetated patches only close to roads, and they selected, or at least were neutral to, such sites if they were far from roads. This could be because the risk of being disturbed was lower there. However, note that areas outside densely vegetated patches and far from roads are rare in the landscape, leading to large confidence intervals that warrant caution in interpretation. Wild boars' response to the proximity of roads changed during the HS, as they selected densely vegetated sites more strongly when they were close to roads and did not select sites in more open vegetation even far from roads. We argue that this could reflect a proactive strategy of wild boars to minimize hunting risk. Hunters tend to focus their effort away from main roads as they make hunting dangerous for people and dogs. Areas near roads are thus safer than elsewhere, and exposure in the open, far from roads is likely particularly unsafe for a wild boar during the HS.

Resting strategies that involve behavioral adjustments to the risk of disturbances by people, as demonstrated here for wild boars, have been documented before (Bojarska et al., 2021; Scillitani et al., 2010; Wittemyer et al., 2017). Previous studies, however, rarely evaluated how successful these adjustments were at reducing this risk. We addressed this gap by studying the probability of wild boars relocating during their resting phases, a possible indicator of disturbance, depending on the location of the RSs. Our results generally suggest that the RS selection strategy of wild boars was effective in keeping the probability of being disturbed low, even in places where chances of meeting people is high. Wild boars rested at all distances from villages, and yet the probability of them relocating during the inactive phase was just as low close to villages than several kilometers away from them. This supports our assertion that, close to villages, wild boars use particularly quiet spots where encounters with people are unlikely, and re‐use them over time. They could thus rest in anthropized landscape with perhaps little cost.

Of course, not all disturbances may lead to a relocation. It is well known that wild boars may hide rather than flee when hunted (Scillitani et al., 2010; Thurfjell et al., 2013), and wild boars may sometimes be disturbed and not leave their RS. We actually observed this response in 62% (8 out of 13) of the experimental disturbances that we led on resting wild boars (personal observations). Although any disturbance can affect the “quality” of resting (e.g., through sleep deprivation), keeping quiet in response to an untargeted disturbance is likely a good strategy, as it avoids the costs and risks associated with relocating. Besides, non‐targeted disturbances are often short‐lived, and will only reduce resting time marginally. Additionally, in our study, the wild boars remained inactive for 14 h/day on average, which was longer than the total sleep time previously recorded in domestic pigs (7.8 h; Campbell & Tobler, 1984) and in free‐ranging wild boars (10.6 h; Mortlock et al., 2022). This suggests that the wild boars were unlikely to suffer harmful reductions in resting time due to anthropic disturbances. In some cases, wild boars might not even respond to a stimulus that we would have assumed stressful for them. For instance, upon opportunistically listening to the rest of the audio data, we heard a dog barking from what seemed like a close distance, but the wild boar did not react and continued to snore calmly throughout the event (Appendix S4). This extract supports the hypothesis that resting wild boars show little sensitivity to untargeted disturbances. Given the low frequency of relocations, and the little impact expected from disturbances that do not entice relocations, it seems safe to conclude that wild boars, in our study area, are little affected by human activity during resting. Unsurprisingly though, this was less true during the hunting season as hunting is a significant source of targeted disturbance for wild boars in this landscape. The probability of relocation was highest during hunting days of the HS, and long‐distance relocations (>1 km) were virtually only observed during these days. These could correspond to actual hunts, with dogs chasing the animal over large distances. The moderately greater probability of relocation in non‐hunting days of the HS might reflect a higher responsiveness of wild boars to even non‐targeted disturbances, during the HS. However, it is important to note that the low explanatory power of the models suggest that the fact that the hunting season was open did not substantially change the causes or consequences of relocations, and therefore the relocation patterns of wild boars. It likely only added the possibility that a hunting event would sometimes cause some long‐distance relocation. Of course, not all relocations may have been a response to a disturbance. In 9 out of 10 audio extracts collected at the onset of a relocation, the animal could only be heard resting quietly and snoring, before leaving the area for no apparent reason. It is possible that we did not hear a person or a dog that was nearby, which the wild boar would have smelt, or heard, and reacted to. It is also possible that the wild boar simply woke up and left for a new area for a different reason (e.g., weather change). If such natural daily movement occur during the inactive phase, this would strengthen our general interpretation that people rarely disturb wild boars once they have selected a RS.

In conclusion, our study shows that wild boars are efficient at exploiting areas near people, while still finding suitable sites where to rest. They do so by selecting sites with high cover, by responding to the proximity of human presence in ways that can change with the hunting season, and by revisiting preferentially the safest sites near human settlements. It is apparently a successful strategy, as they rarely have to relocate far away in response to a disturbance, apart when targeted by hunting. As more and more habitats become subject to anthropization, the need to find suitable sites where to rest is likely to become a constraint for a variety of animal species. In such circumstances, species with a high behavioral and ecological flexibility are likely to be favored, in particular through the adjustment of their resting patterns. Although successful temporal adjustments of activity patterns in response to human pressure had been extensively described (Gaynor et al., 2018; Lowry et al., 2013), our study shows that resting site selection is another efficient way for animals to avoid encounters with people.

## AUTHOR CONTRIBUTIONS

**Gustave Fradin:** Conceptualization (equal); data curation (lead); formal analysis (lead); investigation (supporting); visualization (lead); writing – original draft (lead); writing – review and editing (equal). **Simon Chamaillé‐Jammes:** Conceptualization (equal); formal analysis (supporting); funding acquisition (lead); investigation (lead); project administration (lead); supervision (lead); writing – review and editing (equal).

## CONFLICT OF INTEREST STATEMENT

The authors declare no conflict of interest.

## Supporting information


Figure S1

Figure S2

Figure S3

Figure S4

Appendix S1‐S4

## Data Availability

GPS and activity data collected on wild boars are available at https://doi.org/10.5061/dryad.3j9kd51qj.
