# Supplementary figures and images for: Hogs sleep like logs: Wild boars reduce the risk of anthropic disturbance by adjusting where they rest
Source: Ecol Evol. 2023 Jul 22;13(7):e10336. doi: 10.1002/ece3.10336 (PMC10363780; doi:10.1002/ece3.10336)

(a)

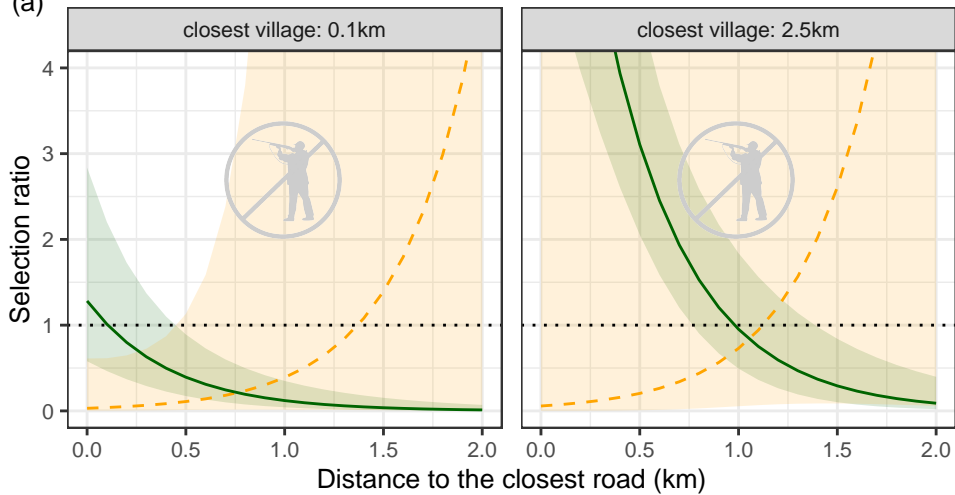

(b)

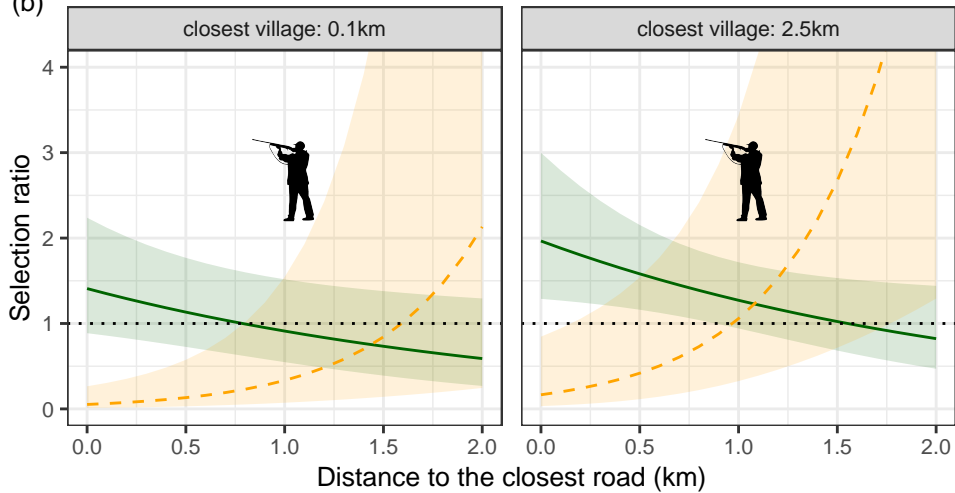

dense vegetation patches

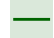

inside

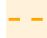

outside

Supplement: Supplementary file 1 — Figure S1 [file ECE3-13-e10336-s003.pdf]

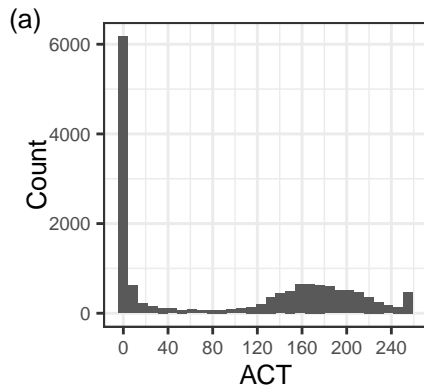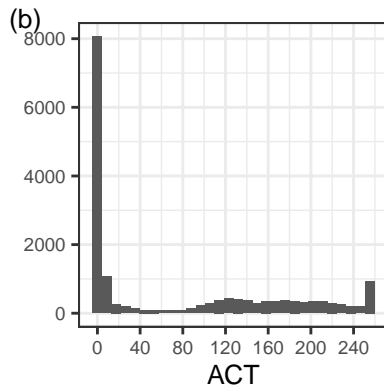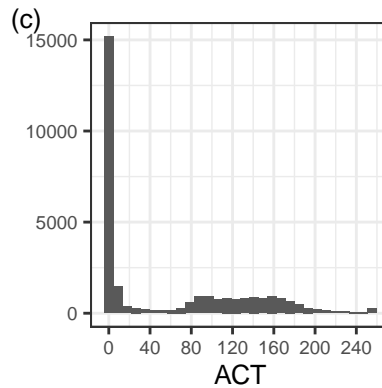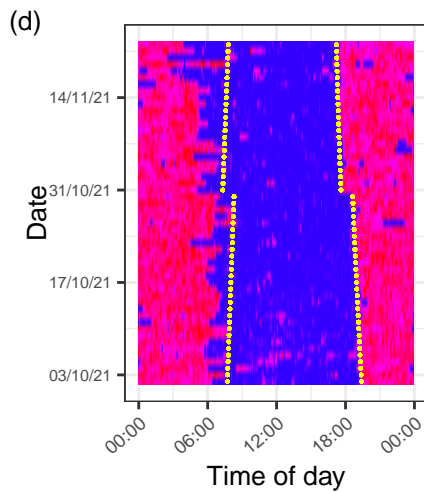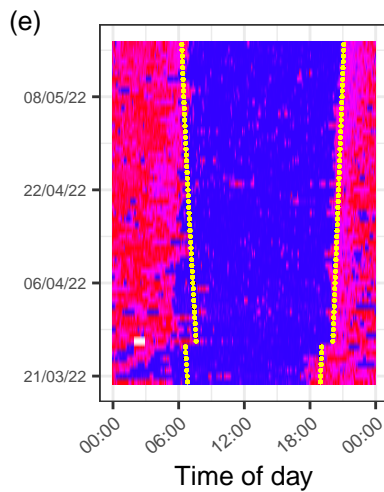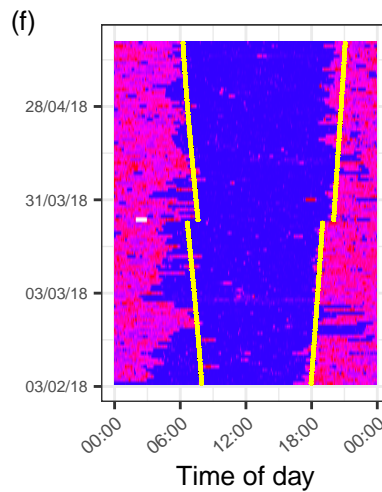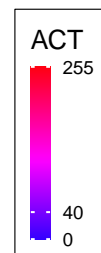

Supplement: Supplementary file 2 — Figure S2 [file ECE3-13-e10336-s004.pdf]

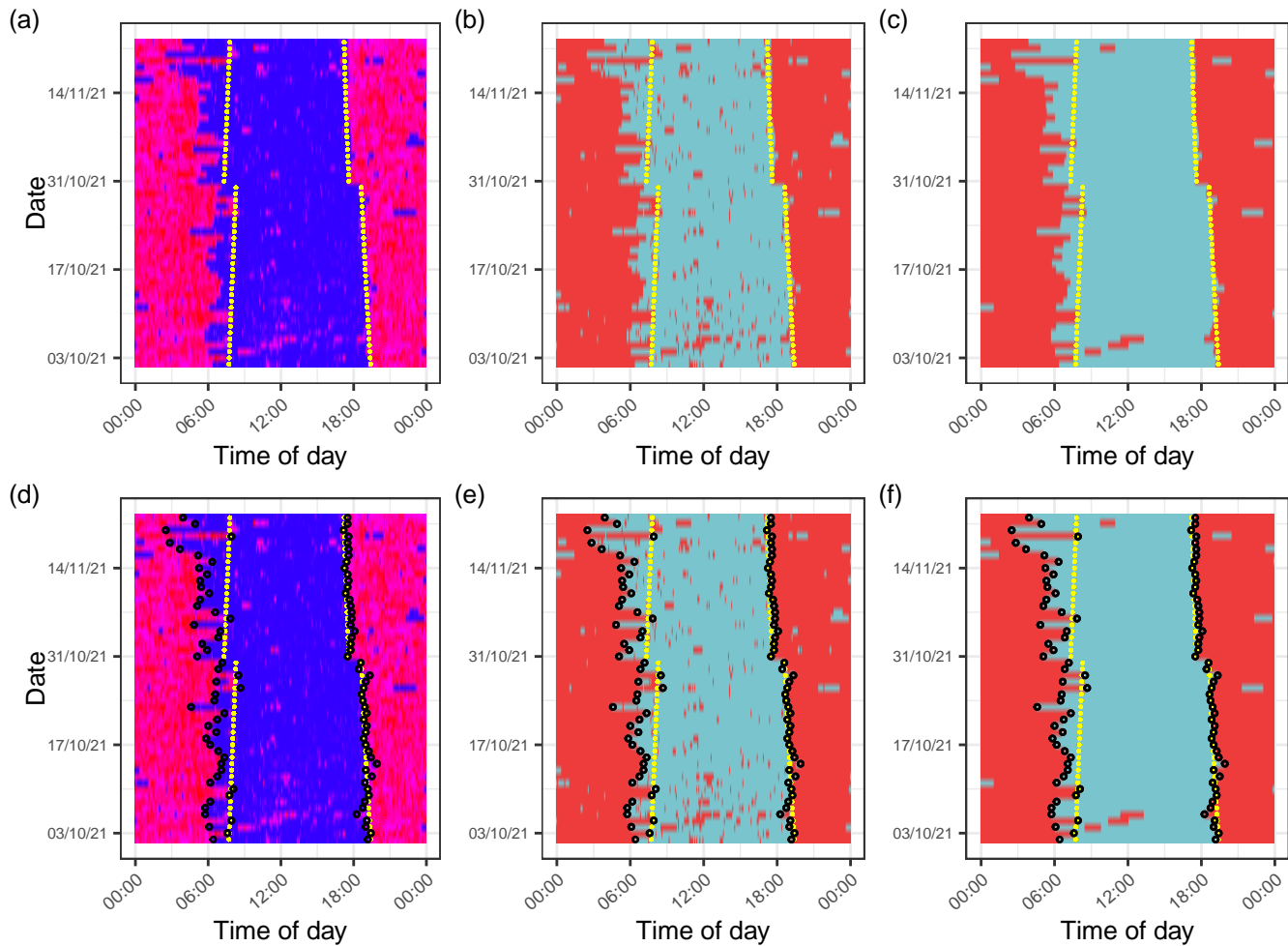

Supplement: Supplementary file 3 — Figure S3 [file ECE3-13-e10336-s005.pdf]

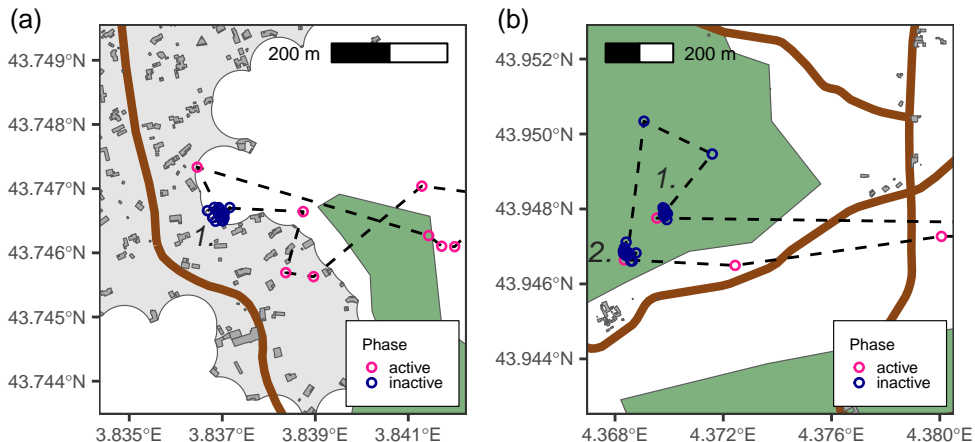

(c)

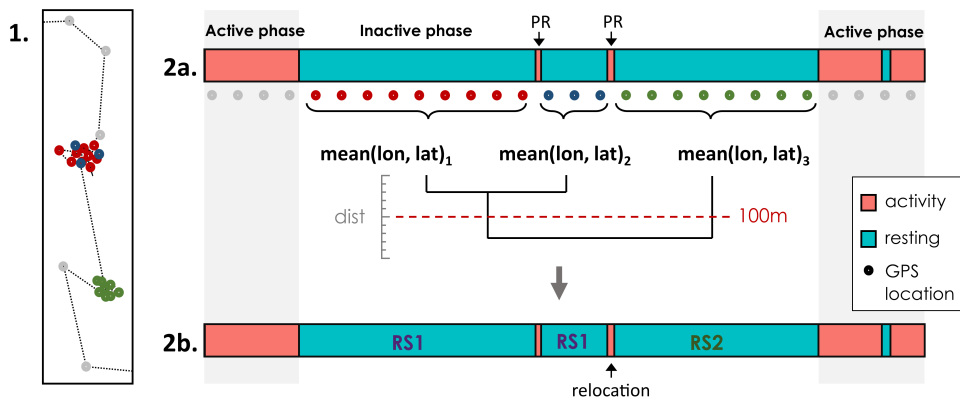

Supplement: Supplementary file 4 — Figure S4 [file ECE3-13-e10336-s001.pdf]
