# Supplementary material for: Hogs sleep like logs: Wild boars reduce the risk of anthropic disturbance by adjusting where they rest
Source: Ecol Evol. 2023 Jul 22;13(7):e10336. doi: 10.1002/ece3.10336 (PMC10363780; doi:10.1002/ece3.10336)
Supplement: Supplementary file 5 — Appendix S1‐S4 [file ECE3-13-e10336-s002.docx]

## Appendix S1. Selection of resting sites by the females

**We could collect only very limited data from female wild boars. We tracked only five females, of which two did not provide data during the NHS. They** rested mostly inside densely vegetated patches, and extremely few resting sites were located out of them: nine during the HS and two during the NHS. In addition, **few resting sites were located far from a road or close to a village.** It is well known that male and female wild boars generally differ in their use of space, habitats, and responses to anthropic pressures (Saïd et al. 2012; Morelle et al. 2015). Considering that, it did not seem relevant to include the females in the analysis without accounting for the effect of sex. **Fitting models with the data from both sexes and considering sex as a fixed effect was impossible, due to the low amount of data from females. This is why we fitted models on the males’ and the females’ data separately. Here we provide information about the exploration of the females’ data.**

**We used the method described in section 2.4.1 of the main text to fit a resource selection function (RSF) to the females’ data. As with the males, we fitted a GLMM, using the type of resting site (used vs. available) as a response variable and a binomial distribution for errors. We used the vegetation cover (inside or outside densely vegetated patches) in interaction with the distances to roads and to villages as predictor variables. We also included individual animal identities nested within study sites as random intercepts. As with the males, we fitted this model independently for the HS and the NHS.** **Finally, we converted RSF scores into selection ratios for interpretability.**

During the NHS, the lack of data made it impossible to draw any conclusion on the selection of resting sites by females. We fitted the model on only three individuals, with two resting sites observed outside densely vegetated patches and two observed further than a kilometer from a road. This explains the large confidence intervals outside densely vegetated patches, as well as the avoidance that we observed far from roads (Figure S1a, Table S1).

During the HS, more data was available, although the overall amount remained limited. The results were similar to those obtained for the males (Figure S1b, Table S1). The selection ratio for sites outside densely vegetated patches was globally similar to the one observed for the males. However, we found a significant effect of the interaction between the type of vegetation and the distance to roads, while it was not significant in males. Once again, however, the low amount of data for females outside densely vegetated patches made it complicated to interpret this result.

| **Variable** | **ß** | **SE** | **z-value** | **p-value** |
| --- | --- | --- | --- | --- |
| **Model: Selection ratio for resting sites during the non-hunting season (females)** | | | | |
| Intercept | -10.42 | 1.56 | -6.70 | <0.001 |
| inside densely vegetated patches | 3.68 | 1.61 | 2.28 | 0.02 |
| d(road) | 2.55 | 2.18 | 1.17 | 0.24 |
| d(village) | 0.26 | 1.00 | 0.26 | 0.79 |
| inside densely vegetated patches x d(road) | -4.91 | 2.23 | -2.20 | 0.03 |
| inside densely vegetated patches x d(village) | 0.60 | 1.02 | 0.59 | 0.56 |
| **Model: Selection ratio for resting sites during the hunting season (females)** | | | | |
| Intercept | -9.91 | 0.87 | -11.45 | <0.001 |
| inside densely vegetated patches | 3.33 | 0.90 | 3.70 | <0.001 |
| d(road) | 1.85 | 0.58 | 3.18 | <0.01 |
| d(village) | 0.48 | 0.50 | 0.97 | 0.33 |
| inside densely vegetated patches x d(road) | -2.29 | 0.62 | -3.71 | <0.001 |
| inside densely vegetated patches x d(village) | -0.34 | 0.51 | -0.66 | 0.51 |
| “d(village/road)” stands for “distance to the closest village/road.” | | | | |

**Table S1.** Parameters and statistics for the models estimating the selection ratio for resting sites by females, during the non-hunting season and the hunting season. The 5-fold cross-validation performance scores (Spearman-rank correlations) of the models are respectively 0.58 ± 0.18 s.e. and 0.53 ± 0.13 s.e.

## Appendix S2. Identification of resting patterns

1. Identification of the inactive phases

From visual inspection of the activity data, we inferred that, in our study area, the wild boars were nocturnal. The distribution of activity values (ACT) was bimodal for all individuals, with one mode around zero - corresponding to resting - and one mode between 80 and 240 - corresponding to activity (three examples in Figure S2a-b-c). We chose a threshold value (ACT=40) to separate those activity values in two groups such that the least frequent values of activity, i.e., those between 10 and 80, were mostly classified as “activity.” We thus kept the “resting” category as constrained as possible. All the individuals considered in the analysis showed a clear nocturnal pattern of activity, with one sequence of low values of activity spanning throughout daytime - sometimes starting a little before sunrise - and one sequence of high values of activity spanning quite precisely from sunset to the start of the next sequence of inactivity, in the morning (three examples in Figure S2d-e-f).

Based on the observed temporal patterns of activity, we considered that each 24-hour cycle could be divided into two continuous phases: the active and the inactive phase. We identified the time boundaries of these phases by applying the same treatment to each animal’s activity data. We first applied a threshold on the raw activity data (one example in Figure S3a) to obtain a binary time series with values being either “active” (ACT>40) or “resting” (ACT≤40) behavior, each value corresponding to a 5min time slot (Figure S3b). Many short sequences of “active” behavior were typically spread across what would be the inactive phase, i.e., during the day, and conversely, some short “resting” sequences existed within what would be the “active” phase, i.e., during the night. We therefore simplified the time series by smoothing out the short sequences that we considered irrelevant for the determination of the time boundaries of “active” and “inactive” phases. We did so by iteratively switching the category (“resting” or “active”) of the shortest sequence in the time series, until the shortest sequence in the entire time series was 1 hour long (Figure S3c). Five minutes time slots with intermediate activity values (between 10 and 80) were rarely consecutive. For that reason, the outcome of the last processing step was robust to the choice of a different threshold value to identify “active” and “resting” behaviors. Finally, we determined the times of the starting and ending of the “inactive” and “active” phases, so that they matched as closely as possible the pattern observed visually on the raw data (Figure S3d, E and F). We used the following three rules to attribute each sequence of the time series to one or the other phase: (i) All sequences of “resting” behavior overlapping daytime, and (ii) all sequences of “active” behavior spanning entirely within daytime were considered part of the inactive phase. In addition, (iii) whenever a sequence of “resting” behavior restricted to nighttime lasted more than 1.5h, and was separated from the rest of the “inactive” phase by an “active” sequence less than 1.5h long, both the “resting” and the “active” sequences were added to the “inactive” phase. The result of this last step closely matched the pattern expected by visual inspection of the raw data (Figure S3d).

2. Identification of the resting sites

After having determined the start and end times of the daily inactive phases, we used GPS data to identify where resting had occurred each day. Often, resting occurred at a single “resting site” (RS) throughout the inactive phase (example in Figure S4a). In some instances, however, wild boars did move from an initial RS, to settle in a secondary RS, during the inactive phase, in what we called a “relocation” (example in Figure S4b). Relocations would obviously be associated with important values of ACT. To identify them, we compared the location of the animal before and after the bouts of activity that we considered sufficiently “active” to be considered a “possible relocation” (hereafter PR, see Figure S4c). We considered a PR whenever a continuous “active” bout reached a cumulative ACT value of at least 100. Between PRs, the ACT signal was too low for the animal to have possibly traveled from one RS to another. We compared the mean GPS positions before and after the first PR of each inactive phase. If they were further apart than 100m, we considered that a relocation had occurred. If not, we discarded the PR and applied the same method to the next PR in the inactive phase. Once we had identified all the relocations, we estimated the exact location of the different RSs to be the average of the GPS positions recorded during the inactive phase, between each relocation (Figure S4c).

## Appendix S3. Identification of densely vegetated patches

We retrieved spatial information on vegetation cover from CORINE Land Cover 2018 (https://land.copernicus.eu/pan-european/corine-land-cover/clc2018). The following list shows the categories that we included in the definition of “densely vegetated patches”, together with the percentage of the land they occupied in our study area:

- Broad-leaved forest (14.5%)

- Coniferous forest (2.4%)

- Mixed forest (4.4%)

- Sclerophyllous vegetation (12.7%)

- Transitional woodland-shrub (4.1%)

- Land principally occupied by agriculture, with significant areas of natural vegetation (3.6%)

- Natural grasslands (2.2%)

- Moors and heathland (0.3%)

Although usually associated with low vegetation, the “natural grasslands” and the “moors and heathland” categories corresponded, in our study area, to areas of regrowth, offering suitable vegetation cover for wild boars to hide. This is why we considered relevant to group them together with taller vegetation layers in this study.

## Appendix S4. Sound extracts

All the sound extracts mentioned in the paper are available for listening at the following address: https://doi.org/10.5281/zenodo.7660803. The tracks that were extracted from 40 minutes before to 5 minutes after a relocation was initiated are labelled as “relocation.” The tracks labelled as “control” were extracted at the same time of day as the “relocation” tracks, but on different dates, when relocations did not occur. We also make available an additional track, corresponding to a dog barking, with the wild boar not reacting to it. It was identified upon opportunistic listening of the dataset.

## Legends of the figures of the appendices

**Figure S1.** Estimation of the selection ratio for resting sites by female wild boars during the non-hunting season (a) and the hunting season (b), for different distances to roads and villages. Selection for densely vegetated patches is represented in a dark green, solid line. Selection for non-densely vegetated patches is represented in a yellow, dashed line. A selection ratio above one indicates that sites are selected, and a selection ratio below one indicates that sites are avoided. Lightly-colored ribbons show the 95% confidence intervals.

**Figure S2.** Patterns of activity of three wild boars. (a-b-c) Distribution of the raw ACT values, for three individuals from our study. (d-e-f) Temporal patterns of activity of the animals presented respectively in (a), (b) and (c). Each date (by rows) is divided into 5min slots that are colored according to their level of activity, ranging from blue (ACT=0) to red (ACT=255). The yellow dots indicate, for each date, the times of sunrise and sunset. Times are in local time, which includes daylight savings (note the discontinuities in sunrise and sunset times).

**Figure S3.** Visual description of the steps taken to determine the timing of the “inactive” phases. (a) Temporal pattern of activity of one wild boar, as raw data. The scale used is the same as in Figure S2. (b) Result of the application of a threshold on the time series of raw data, to distinguish “active” (ACT>40, in red) and “inactive” (ACT<=40, in light blue) behaviors. (c) Result of the smoothing processing step applied to the time series shown in (b). (d), (e) and (f) respectively show how the start and end times of the “active” and “inactive” phases (black circles) fit on the time series presented in (a), (b) and (c). The yellow dots indicate, for each date, the times of sunrise and sunset.

**Figure S4.** Identification of the relocations. (a) Trajectory of a wild boar (dashed line), including an inactive phase with no relocation, and thus a single resting site (1). (b) Trajectory of a wild boar (dashed line), including an inactive phase with a relocation, in which the animal traveled from a primary resting site (1) to a secondary resting site (2). In (a) and (b), buildings are represented in dark grey, villages in light grey, roads in brown, and densely vegetated patches in dark green. (c) Method of identification of the relocations. We focus on a theoretical trajectory, spanning across one inactive phase, and represented spatially in (1). The associated time series of “active” and “resting” bouts is represented in (2a). The colors of the dots indicate groups of GPS locations that were acquired during the same resting bout. During the inactive phase, between the potential relocations (PR), the distances (dist) between mean GPS positions (mean(lon, lat)) are calculated. Whenever dist exceeds 100m, we consider that the PR corresponds to a relocation. This method enables the identification of RS1 and RS2, two resting sites that were visited during the inactive phase (2). The first PR did not correspond to a relocation, but the second one did.
